# Commentary: Inflammatory and Oxidative Responses Induced by Exposure to Commonly Used e-Cigarette Flavoring Chemicals and Flavored e-Liquids without Nicotine

**DOI:** 10.3389/fphys.2018.01240

**Published:** 2018-09-13

**Authors:** Massimo Caruso, Giovanni Li Volti, Pio Maria Furneri, Virginia Fuochi, Rosalia Emma, Riccardo Polosa

**Affiliations:** ^1^Department of Clinical and Experimental Medicine, University of Catania, Catania, Italy; ^2^Department of Biomedical and Biotechnological Sciences, University of Catania, Catania, Italy; ^3^Center of Excellence for the Acceleration of Harm Reduction, University of Catania, Catania, Italy

**Keywords:** e-Cigarette, flavoring agents, inflammation, oxidative stress, e-liquids

Muthumalage et al. (2017) have recently investigated the effects of a range of flavoring chemicals and flavored e-liquids on two monocytic cell lines, MM6 and U937. The authors have shown that by exposing monocytes to flavorings used in e-liquids it is possible to elicit a cytotoxic as well as an inflammatory response mediated by ROS production and conclude that this may provide insights into potential inhalational risk of e-cigarette use.

There is a tendency to exaggerate potential health risks of e-cigarettes with little or no consideration for the emerging health benefits. The current study is no exception. In particular, translating the study's findings into a real-life setting is questionable.

First, no specific information on the regime used to generate the aerosol was provided; in particular no details on device, voltage, puff volume, puff duration, and puffing profile were reported.

Second, biologic and toxicological responses are normally expected when cells are chronically and continuously exposed to chemicals at high concentrations. Unsurprisingly, cytotoxic as well as non-specific inflammatory and oxidative stress responses were shown in monocytic cell lines exposed for no less than 24 h to (some) chemical flavorings at high concentrations and to a mix of flavorings-containing e-liquids. Furthermore, important consideration must be given to the fact that flavorings gets rapidly degraded in the blood. For example, cinnamaldehyde is oxidized very rapidly to cinnamic acid (Bickers et al., 2005) in rat as well as in humans (Quarto di Palo and Bertolini, 1961; Yuan et al., 1992; Joint Expert Committee on Food Additives, 2000). In a previous study, Yuan et al. determined that the maximum concentration of cinnamaldehyde in the blood reach 7.6 μM after a 250–500 mg/Kg oral dose in rats (Yuan et al., 1992). Muthumalage et al. (2017) exposed monocytes to a range of concentration between 10 and 1000 μM, which is higher than the maximum reported in rats. Moreover, less than 0.1% of cinnamaldehyde remains in the blood, with a high-life ranging from minutes to 2 h (Quarto di Palo and Bertolini, 1961; Lee et al., 2009). So, the exposure system used by Muthumalage et al do not take in account the accelerated metabolism of flavorings and likely to overemphasize their harmful effects. Chronic exposure to high levels of sugar or salt in a water solution would have triggered similar responses (Garland et al., 1989). Moreover, authors exposed monocytes to flavoring chemicals at a range concentration from 10 to 1000 μM when the major international agencies report limits of exposure that are significantly lower (Table 1).

**Table 1:** Exposure limits set by the major safety agencies for following chemical flavorings.

**Chemical Flavoring**	**CAS N^°^**	**Agency**	**Human exposure**	**Ref**.
Diacetyl **(2,3-Butanedione)**	431-03-8	NIOSH REL	0.005 ppm (0.12 μM) Occupational exposure	NIOSH 2011
		NIOSH STEL	0.025 ppm (0.35 μM) Occupational exposure	
		ACGIH TLV	0.01 ppm (0.12 μM) Occupational exposure	ACGIH 2014
Cinnamaldehyde (3-Phenylprop-2-enal)	104-55-2	EFSA	97.4 μg/kg bw per day (0.76 μM)	EFSA FEEDAP Panel, 2017
		ECHA	NCL	REACH 2018
Acetoin (3-Hydroxy-2-butanone)	51555-24-9	NIOSH REL	NA	NIOSH 2011
		NIOSH STEL	NA	
		ECHA	NA	REACH 2018
Maltol (3-Hydroxy-2-methyl-4-pyrone)	118-71-8	EFSA	166 μg/kg bw per day (1.29 μM)	EFSA FEEDAP Panel, 2016
		ECHA	NCL	REACH 2018
Acetylpropionyl (2,3-pentanedione)	600-14-6	NIOSH REL	0.0093 ppm (0.10 μM) Occupational exposure	NIOSH 2011
		NIOSH STEL	0.031 ppm (0.30 μM) Occupational exposure	
o-vanillin **(2-Hydroxy-3-methoxybenzaldehyde)**	121-33-5	EFSA	1 g/kg bw per day	EFSA ANS Panel, 2018
		ECHA	NCL	REACH 2018
Coumarin (1-benzopyran-2-one)	91-64-5	EFSA	0.1 mg/kg bw per day (6.85 μM)	EFSA 2008
		ECHA	DL	REACH 2018

NIOSH, National Institute for Occupational Safety and Health; ACGIH, American Conference Of Governmental Industrial Hygienists; ECHA, European Chemicals Agency; EFSA, European Food Safety Authority;

*NA, not available; NCL, no classification limit; REL, recommended exposure limit; STEL, short-term exposure limit; TLV, threshold limit value; DL, data lacking*.

Third, the reported effects are observed when monocytes are in direct contact with flavoring chemicals. Such *in vitro* experimental set up does not resemble normal condition of exposure in e-cigarette users, because e-liquids containing flavoring chemicals are vaporized before entering in contact with circulating monocytes in the human body, with considerable losses of flavoring substance, sometimes. The loss of flavors caused by the system generating aerosol has been clearly demonstrated in a recent work that dosed the concentration of flavors in e-liquids and in vapor e-liquids condensate with gas-chromatography coupled to mass spectrometry (Clapp et al., 2017), evidencing a mean loss of Cynnamaldheyde content in aerosolized e-liquids of 58,67% (ranging from 17.19 to 83.75%).

Fourth, in addition to the reduced concentration of flavors due to endogeneous catabolisms and the vaporization process, it must be considered that the remaining flavors must overcome the physiological barrier consisting of the airway epithelium, before getting to reach the monocytes. The airway epithelium forms the first continuous line of defense, able to dynamically regulate its response to experienced luminal stimuli, against inhaled environmental insults, which include pathogens, pollutants, chemicals and aeroallergens (Brune et al., 2015). So, flavorings levels that will eventually come into contact with circulating monocytes will be just a fraction of those used in this investigation. The exposure condition reproduced in the study is more similar to that of an intravenous infusion with circulating monocytes being exposed to very high levels of chemical flavorings; once again not a situation resembling normal condition of e-vapor exposure in humans.

Fifth, even if we accept that flavoring chemicals have detrimental effects on monocytic cell lines, it must be noted that these findings are clinically irrelevant and without prognostic value for the health of e-cigarette users. The positive evidence from real-life surveys and clinical studies of patients with respiratory conditions supporting respiratory health benefits with e-cigarette use (Polosa et al., 2014, 2016a,b) and from a cohort of long-term daily e-cigarette users (>3.5 years) who have never smoked in their life showing no indication of emerging lung injury as reflected in physiologic, clinical, radiologic, and inflammatory measures (Polosa et al., 2017) is in stark contrast with the concerns raised in experimental models.

In conclusion, because the experimental protocol is designed to elicit biologic as well as toxicological responses, the study findings overestimate the health concern associated with the exposure to some flavorings. Flavorings at high concentrations are known to cause local irritative effects and non-specific inflammation that are usually transient and reversible. Besides, the human body is equipped with extremely efficient detoxification and scavenging systems that would take care of the exposure to potentially harmful chemicals. Most importantly, findings of the current study fail to add to our understanding of the risks of these products, as the experimental conditions described by the Authors fail to replicate normal condition of use/exposure. It is therefore urgent to address common mistakes and to develop robust and realistic methodological recommendations in order to adequately assess the impact of e-cigarette use on human health under normal condition of use. Adoption of standardized methods will also enable a better understanding, comparison and extrapolation of results obtained across various studies and research groups.

## Author contributions

MC, PMF, GL, and RP have made substantial contributions to the conception of the work. MC and RE have drafted the work. PMF and VF have made contribution in interpretation of data from originally work. All the authors provided approval for publication of the content.

### Conflict of interest statement

The authors declare that the research was conducted in the absence of any commercial or financial relationships that could be construed as a potential conflict of interest.
